# Glycan masking of a non-neutralising epitope enhances neutralising antibodies targeting the RBD of SARS-CoV-2 and its variants

**DOI:** 10.3389/fimmu.2023.1118523

**Published:** 2023-02-23

**Authors:** George W. Carnell, Martina Billmeier, Sneha Vishwanath, Maria Suau Sans, Hannah Wein, Charlotte L. George, Patrick Neckermann, Joanne Marie M. Del Rosario, Alexander T. Sampson, Sebastian Einhauser, Ernest T. Aguinam, Matteo Ferrari, Paul Tonks, Angalee Nadesalingam, Anja Schütz, Chloe Qingzhou Huang, David A. Wells, Minna Paloniemi, Ingo Jordan, Diego Cantoni, David Peterhoff, Benedikt Asbach, Volker Sandig, Nigel Temperton, Rebecca Kinsley, Ralf Wagner, Jonathan L. Heeney

**Affiliations:** ^1^ Lab of Viral Zoonotics, Department of Veterinary Medicine, University of Cambridge, Cambridge, United Kingdom; ^2^ Institute of Medical Microbiology & Hygiene, Molecular Microbiology (Virology), University of Regensburg, Regensburg, Germany; ^3^ DIOSynVax, Ltd., Cambridge, United Kingdom; ^4^ Applied Science & Technologies, ProBioGen AG, Berlin, Germany; ^5^ Viral Pseudotype Unit, Medway School of Pharmacy, The Universities of Kent and Greenwich at Medway, Chatham, United Kingdom; ^6^ Institute of Clinical Microbiology and Hygiene, University Hospital Regensburg, Regensburg, Germany

**Keywords:** SARS-CoV-2 antibody, receptor binding domain (RBD), glycan masking, neutralising antibodies, pseudotype neutralisation

## Abstract

The accelerated development of the first generation COVID-19 vaccines has saved millions of lives, and potentially more from the long-term sequelae of SARS-CoV-2 infection. The most successful vaccine candidates have used the full-length SARS-CoV-2 spike protein as an immunogen. As expected of RNA viruses, new variants have evolved and quickly replaced the original wild-type SARS-CoV-2, leading to escape from natural infection or vaccine induced immunity provided by the original SARS-CoV-2 spike sequence. Next generation vaccines that confer specific and targeted immunity to broadly neutralising epitopes on the SARS-CoV-2 spike protein against different variants of concern (VOC) offer an advance on current booster shots of previously used vaccines. Here, we present a targeted approach to elicit antibodies that neutralise both the ancestral SARS-CoV-2, and the VOCs, by introducing a specific glycosylation site on a non-neutralising epitope of the RBD. The addition of a specific glycosylation site in the RBD based vaccine candidate focused the immune response towards other broadly neutralising epitopes on the RBD. We further observed enhanced cross-neutralisation and cross-binding using a DNA-MVA CR19 prime-boost regime, thus demonstrating the superiority of the glycan engineered RBD vaccine candidate across two platforms and a promising candidate as a broad variant booster vaccine.

## Introduction

Severe acute respiratory syndrome coronavirus 2 (SARS-CoV-2) is the causative agent of COVID-19. Since its emergence in late 2019, SARS-CoV-2 has rapidly spread worldwide, causing mortality and morbidity in all age groups, but especially the elderly and those with pre-existing conditions. To date, more than 500 million cases have been reported resulting in around 6.4 million deaths worldwide (1). The first SARS-CoV-2 licensed vaccines expressed a full-length spike based on early WA-1/2020 sequences from the Wuhan outbreak. In phase 3 clinical trials of these vaccines, the key immune marker was neutralising antibody (nAb) responses against SARS-CoV-2 which correlated with protection from hospitalisation, severe disease and mortality (2–9). The vaccine encoded spike protein serves as the most important target antigen as the trimeric spike protein at the virion surface is essential for viral cell entry (10, 11). During infection, SARS-CoV-2 uses the receptor-binding domain (RBD) of the spike protein as a key functional component to interact with angiotensin-converting enzyme 2 (ACE-2) on host cells (12, 13). The trimeric S protein can be in a receptor-inaccessible (closed), or accessible (open) state based on the down or up positions, respectively, of its receptor-binding domain (RBD) (13). Studies have shown that the RBD of SARS-CoV-2 is mainly in the closed conformation which complicates the recognition of the virus particle by the immune system before entering the host cell (14, 15). The receptor-binding motif (RBM) is the most important motif in the RBD and is composed of two regions that form the interface between the S protein and hACE-2. The RBM is responsible for attachment to the ACE-2 receptor. The region outside the RBM is essential in maintaining the structural stability of the RBD (16). Upon RBD-ACE-2 interaction and spike proteolytic priming by the serine transmembrane protease TMPRSS2, conformational changes lead to the membrane fusion of the spike protein and subsequent entry of the virus into the host cell (16). Antibodies targeting the RBD have been reported to be effective against the infection, making RBD subunit-based vaccines a promising candidate for the generation of potent and specific neutralising antibodies (17). Furthermore it was clearly shown that the recombinant spike RBD protein of SARS-CoV-2 can potently induce a protective immune response in mice, rabbits, and non-human primates (18). We propose that modified RBD spike-subunit based booster vaccines may be successful in presenting cryptic neutralising epitopes to recruit additional, broadly neutralising antibodies to epitopes that may otherwise be sub-immunodominant or less accessible if presented as a full-length spike structure. One of the approaches to focus the immune response towards broadly neutralising epitopes is alterations of glycosylation sites, by either removing the glycan sites or introducing new glycan sites. This approach has been described for a variety of viruses such as MERS, SARS-COV-2, and influenza (19–24).

At the beginning of the pandemic, the evolution of SARS-CoV-2 was estimated to be slow, in line with the evolution rate of other human coronaviruses (25). However, since late 2020 numerous SARS-CoV-2 variants of concern (VOCs) with enhanced transmission, pathogenicity, immune escape, or a combination of these attributes have been reported, causing repeated waves of new SARS-CoV-2 infections (26). SARS-CoV-2 VOCs often have mutations arising throughout the genome of the virus, but most of the immune escape mutations are concentrated in the spike protein, especially the RBD. Multiple circulating and evolving lineages of VOCs now exist and were designated by the WHO as Alpha, Beta, Gamma, Delta, and now Omicron variants (27). Many of these variants show an enhanced binding affinity to the human ACE-2 receptor (28–31) in addition to immune escape. Some of the predominant VOC strains identified include those from lineage B.1.1.7 (Alpha), B.1.351 (Beta), P.1 (Gamma), B.1.617.2 (Delta) and B.1.1.529 (Omicron BA.1). B.1.351 and P.1 contain, amongst others, the E484K mutation within the RBD that has been shown to abrogate antibody responses generated from infection or vaccination (3, 32). B.1.617.2 contains the L452R mutation that contributes to immune evasion in combination with T478K, which leads to the increased transmissibility and immune escape seen with this lineage (33, 34). B.1.1.529 has over 30 mutations in the spike protein, influencing neutralising antibodies generated to previous strains or vaccines, as well as reducing the need for TMPRSS2 priming upon viral attachment and entry (35–37). The B.1.1.529 (BA.1) lineage has now antigenically diversified extensively to other sub-lineages such BA.2, BA.4, BA.5 etc. In the light of the continually emerging VOCs there is a need for a new generation of booster vaccines that generate broader neutralising antibodies to provide improved, longer-term protection against current and new emerging SARS-CoV-2 variants.

Towards the development of new and improved SARS-CoV-2 booster vaccines, we developed glycan engineered SARS-CoV-2 RBDs to enhance the breadth of neutralising antibodies to VOCs. To expand the interval between vaccine boosts (currently suggested at 6-to-9-month intervals (38)), we utilised a DNA-prime MVA-boost strategy that is known to be safe and induce long-term immune responses (39). Here we present proof-of-concept data in mice that glycan modified RBD vaccines, delivered as DNA-prime MVA-boost regime, generate potent binding and neutralising antibody response to all the SARS-CoV-2 lineages tested including the RBD from WA-1/2020 strain. The glycan engineered SARS-CoV-2 RBD variant not only demonstrated superior neutralising responses than the wild-type SARS-CoV-2 RBD from WA-1/2020 strain, but it also mitigated virus replication in BALB/c mice following live virus challenge with Australia/VIC01/2020 strain. These data demonstrate the broadly neutralising potential of glycan-engineered SARS-CoV-2 RBD vaccine candidates as future SARS-CoV-2 VOC booster vaccines.

## Methods

### Vaccine design

The epitope regions of CR3022 and S309 were determined using the published structural data in protein data bank (PDB (40–43)) for the antibody complexed with SARS-CoV-2 *viz.* PDB id. 6W41 for CR3022 (41) and PDB id. 6WPS for S309 (42). All the amino acids in antigens that are within 5Å contact of amino acids of antibodies were considered as epitope residues. The position of the glycosylation site was determined by *in-silico* mutation of triplets of amino acids in the epitopes to glycosylation sequon – N-X-T (44) using the FoldX algorithm (43). Briefly, residues succeeding N-X motif, where X can be any amino acid except Pro, were mutated to either Threonine or Serine or residues preceding X-T, where X can be any amino acid except Pro, were mutated to Asn to generate novel N-X-T/S motifs. The mutations with the least energy cost, as calculated by the Build module of FoldX, were selected for designing M7 and M8.

### Cells

HEK293T cells and DF-1 cells were maintained and grown in Dulbecco’s MEM (DMEM) supplemented with 10% Fetal Calf Serum (FCS) and 1% Penicillin/Streptomycin (Pen/Strep) at 37°C and 5% CO_2_ in a humidified incubator. For the generation of recombinant MVAs the AGE1.CR.pIX cell line was used (ProBioGen AG, Berlin). Adherent AGE1.CR.pIX cells were maintained in DMEM-F12 medium supplemented with 5% bovine serum (γ-irradiated, Sigma Aldrich/Merck, 12003C) and 2 mM GlutaMAX I (10565-018)).

### Production of lentiviral pseudotypes

Lentiviral pseudotypes were produced by transient transfection of HEK293T/17 cells with packaging plasmids p8.91 (45, 46) and pCSFLW (47) and different SARS-CoV-2 VOC spike-bearing expression plasmids using the Fugene-HD transfection reagent (48, 49). Supernatants were harvested after 48h, passed through a 0.45 µm cellulose acetate filter and titrated on HEK293T/17 cells transiently expressing human ACE-2 and TMPRSS2. Target HEK293T/17 cells were transfected 24h prior with 2 µg pCAGGS-huACE-2 and 75 ng pCAGGS-TMPRSS2 (16, 50).

### Live virus for challenge study

For live virus experiments and challenge the strain Australia/VIC01/2020 was used (51), representing one of the early strains of SARS-CoV-2 B type. Australia/VIC01/2020 was obtained from the University of Melbourne through Public Health England.

### Antigen sequences and generation of DNA expression vectors

The sequence encoding the full-length SARS-CoV-2 spike gene (EPI_ISL_402119) and the SARS-CoV-2 RBD variants M7 and M8 were codon optimised and synthesised by Geneart/Thermo Fisher (Regensburg, Germany). The antigen encoding the SARS-CoV-2 RBD wt was amplified from the full-length SARS-CoV-2 spike gene by PCR. The antigens expressing SARS-CoV-2 RBD wt, RBD M7 and RBD M8 were cloned into the DNA expression vector pURVac *via* restriction digestion. The pURVac DNA expression vector includes a strong human cytomegalovirus (CMV) promoter to initiate transcription of the encoded antigen in combination with a human T-cell leukaemia virus-1 (HTLV-1) regulatory element and a bovine growth hormone poly-A terminator. Sequences were verified using Sanger Sequencing. The DNA vaccine vectors were purified using the EndoFree Plasmid Mega kit (Qiagen, Hilden, Germany) according to the manufacturer’s instructions.

### Protein production

DNA plasmids expressing SARS-CoV-2 RBD wt and the various SARS-CoV-2 RBD VOC variants were cloned into a modified pcDNA5/FRT/TO encoding a minimal N-terminal tPA signal peptide and a C-terminal avi-hexahistidine tag. Proteins were expressed and purified essentially as described earlier (52). In brief, DNA plasmids encoding the above RBD variants were transfected into Expi293 cells (Thermo Fisher Scientific) using the commercial ExpiFectamineTM protocol according to the manufacturer’s recommendations. Supernatants were harvested after incubation for five days in an orbital shaker (37°C, 8% CO_2_, 90 rpm). For harvesting, the culture was centrifuged twice for 20 min at 1000 x *g* at 4°C. The supernatant was filtered using a 0.22 µm sterile filter and preserved by adding 0.05% (w/v) sodium azide. The soluble RBD protein was purified from the supernatant by immobilised metal ion chromatography (IMAC). The supernatant was loaded twice onto a 5 ml immobilised metal chelate affinity chromatography HisTrap™ Excel column (HisTrap Excel, GE Healthcare) with a flow rate of 1 ml/min followed by recirculation overnight at 4°C using a peristaltic pump. The column was washed with 10 column volumes (50 ml) of 20 mM imidazole in PBS. The protein was eluted by gradually increasing the concentration from 20 mM to 400 mM using a FPLC device (ÄKTA, GE Healthcare). The peak fractions were collected, pooled, and analysed under reducing conditions by SDS (sodium dodecyl sulphate) polyacrylamide gel electrophoresis. For buffer exchange the fractions containing the protein were dialyzed against PBS three times using a Slide-A-Lyzer^®^ 3,5K Dialysis Cassette (Thermo Fisher). For concentrating the protein, the protein solution was added to ultrafiltration devices (Amicon Ultra-15 centrifugal filters, Merck Millipore) with a molecular cutoff of 10 kDa and centrifuged at 4000 rpm.

### Design of MVA shuttle vectors

For the generation of recombinant MVA expressing SARS-CoV-2 RBD wt and RBD M7 the shuttle vectors pMVA Trans TK-SARS-2 RBD wt and pMVA Trans TK SARS-2 RBD M7 were cloned. The MVA shuttle vectors were designed in a way that the antigens SARS-CoV-2 RBD wt and SARS-CoV-2 RBD M7 antigens can be inserted into the thymidine kinase (TK) locus J2R of the parental virus MVA CR19 TK-GFP under the transcriptional control of the early/late modified H5 promoter (mH5) *via* homologous recombination. The MVA shuttle vectors also include the reporter gene β-galactosidase (β-Gal) between the two left arm sequences of the TK locus for screening of recombinant MVAs. After several plaque purification rounds the reporter gene is lost by internal homologous recombination events resulting in a pure (reporter-free) recombinant MVA.

### Generation of the recombinant MVAs

The MVA strain used in this study is MVA CR19 (53, 54). For *in vivo* recombination, adherent AGE1.CR.pIX (1 x 10^6^ cells) were infected with parental MVA CR19 TK-GFP with different MOIs ranging from 0.5 to 0.006, incubated for 2 h, followed by transfection with 0.4 µg of the shuttle vector pMVA Trans-TK-SARS-CoV-2 RBD wt or pMVA Trans-TK SARS-CoV-2 RBD M7 using Effectene (Qiagen, Hilden, Germany) according to the manufacturer’s instructions. After 48 h, the cells were harvested and lysed by three freeze-thaw-cycles and sonication. Pure recombinant MVAs were obtained by sequential agarose plaque purification.

Recombinant MVAs express the SARS-CoV-2 RBD variants and a β-galactosidase reporter gene out of the TK-locus. These viruses were identified and selected for further five plaque purification rounds by staining infected cells with X-Gal (5-bromo-4-chloro-3-indolyl-β-D-galactopyranoside) until no remaining parental MVA-CR19 TK-GFP virus was detected by PCR screening. The coexpressed β-galactosidase reporter gene is placed between two homologous left arm regions of the TK locus. Three additional plaque purification rounds were performed during which the reporter was deleted *via* internal homologous recombination events. The recombinant MVAs encoding SARS-CoV-2 RBD wt or SARS-CoV-2 RBD M7 were plaque purified for another three rounds and the resulting recombinant MVA virus stock was grown on AGE1.CR.pIX cells, purified *via* two ultracentrifugation rounds over a 35% sucrose cushion and titrated on DF-1 cells using crystal violet staining. The sequence of the rMVA and absence of non-recombinant MVA was confirmed using PCR amplification, followed by Sanger sequencing. The expression of SARS-CoV-2 RBD wt and SARS-CoV-2 RBD M7 was confirmed by Western blot analysis in HEK 293 T cells infected with a MOI of 2 and harvested after 24 h.

### PCR analysis of recombinant MVAs

To confirm that the antigen SARS-CoV-2 RBD P521N was inserted correctly into the TK locus of MVA-CR19-TK-GFP, genomic DNA was extracted from infected AGE1.CR.pIX cells in each plaque purification round using the Quick-DNA Miniprep Kit from Zymo Research according to the manufacturer’s instructions. The correct integration into the TK locus was verified by PCR analysis using primers flanking the TK locus. PCR products were separated on a 1% agarose gel, excised, and the expected sequence confirmed by Sanger sequencing.

### Western blot analysis of DNA vaccine vectors and recombinant MVA

For expression analysis by Western blot, cells were lysed in TDLB buffer (50 mM Tris, pH 8.0, 150 mM NaCl, 0.1% SDS, 1% Nonidet P-40, 0.5% sodium deoxycholate) supplemented with protease inhibitors (Complete Mini, Roche, Basel, Swiss). Total protein concentration of the supernatants was determined by Bradford assay (Protein Assay, BioRad, Feldkirchen, Germany). For soluble proteins precipitation with trichloroacetic acid solution was performed as described by Koontz et al., 2014 (55). The proteins were separated on SDS-PAGE under reducing conditions and blotted on a nitrocellulose membrane. The membranes were stained with a primary antibody, the anti-SARS-CoV-2 spike antibody, as a primary antibody. An HRP-labelled secondary antibody and Femto ECL (Thermo Fisher, Waltham, USA) were used for detection in a Chemilux Pro device (Intas, Göttingen, Germany).

### Animal work

8–10-week-old BALB/c female mice (Charles River) were immunised with DNA or MVA constructs bearing the gene of interest, and serial bleeds were taken from the saphenous vein. Terminal bleeds were taken *via* cardiac puncture under isofluorane anaesthesia. For challenge studies, animals were transduced with 1x10^7^ PFU of the ad5-huACE-2 vector in a volume of 75ul by intranasal route (University of Iowa, Viral Vector Core) five days before infection with SARS-CoV-2. Mice were then moved to hermetically sealed isocages at containment level 3 and administered 1x10^4^ PFU of Australia/VIC01/2020 (SARS-CoV-2) by intranasal route under light isofluorane anaesthesia, in a total volume of 40 µl PBS. Animals were weighed and checked twice daily for clinical symptoms and culled on days 3 and 6 post infection by terminal bleed under non recovery anaesthesia. All animal work was approved by the Home Office under project licence P8143424B and approved by the Animal Welfare Ethical Review Body (AWERB). Animal experiments were performed in early 2020 when K18-huACE2 mice colonies were being expanded and were not available.

### RT-qPCR from infected mouse lungs

Mouse lungs frozen in PBS at the time of culling were thawed under containment level 3 conditions and homogenised through a 50 µm cell strainer (Corning) with 1 ml of PBS. The resulting supernatant was centrifuged at 1500 x *g* for 10 minutes, and 140 µl of the supernatant taken for RNA extraction. RNA extraction from the supernatant was performed using Qiamp viral RNA Mini Kit (Qiagen) following the manufacturer’s instructions. 5 µl of the RNA extraction final elution was reverse-transcribed to cDNA and amplified according to the manufacturer’s protocol using TaqMan™ Fast Virus 1-Step Master Mix (ThermoFisher Scientific). The primer pair was as follows: F-5’CAGGTATATGCGCTAGTTATCAGAC-3’ and R-5’CCAAGTGACATAGTGTAGGAATG3’. The probe used was as follows: 5’[6FAM]AGACTAATTCTCCTCGGCGGGCACG[TAM]3’ (Sigma Aldrich). Analysis was performed using the Rotor-Gene 6000 Series Software 1.7 (Corbett Life Sciences, Qiagen). As controls, RNA standards were produced by cloning a 97 nucleotide fragment of the spike open reading frame into the pJET1.2 vector (Invitrogen). This was linearised with HindIII and RNA transcripts made using T7 Ribomax Express Large Scale RNA production 17, under a CC By 4.0 international licence. Transcripts were purified using RNA Clean and Concentrator (Zymo Research) and integrity confirmed by gel electrophoresis.

### Pseudotype based microneutralisation assay

Pseudotype based microneutralisation assay was performed as described previously ^59^. Briefly, serial dilutions of serum were incubated with SARS-CoV-2 spike bearing lentiviral pseudotypes for 1h at 37°C, 5% CO_2_ in 96-well white cell culture plates. 1.5x10^4^ HEK293T/17 transiently expressing human ACE-2 and TMPRSS2 were then added per well and plates incubated for 48h at 37°C, 5% CO_2_ in a humidified incubator. Bright-Glo (Promega) was then added to each well and luminescence was read after a five-minute incubation period. Experimental data points were normalised to 100% and 0% neutralisation controls and non-linear regression analysis performed to produce neutralisation curves and associated IC_50_ values.

### ELISA

Enzyme linked immunosorbent assay was carried out based on published techniques (52, 56). Briefly, 96-well maxisorp plates were coated with SARS-CoV-2 RBD overnight at 1 µg/ml and 50 µl per well. Plates were blocked with 200 µl of 3% non-fat milk with shaking, at room temperature for 1 h. Serum sample dilution series in 1% non-fat milk were performed in dilution plates, and transferred to blocked plates, then incubated for 2h at room temperature with shaking. Plates were then washed three times with PBST and anti-mouse IgG conjugated to horseradish peroxidase (Jackson Immuno, USA) added at a 1:3000 dilution. Plates were incubated for 1h at room temperature, in the dark with shaking. Plates were then washed three times with PBST and 50 µl TMB solution added per well. Reactions were quenched after 2-3 minutes in the dark using 2N H_2_SO_4_, and plates read at 450 nm with a Biorad plate reader. AUC values were generated using Prism GraphPad 9.3.1.

### Data and statistical analysis

All data was processed, and statistical analyses performed using GraphPad Prism 9.2.

## Results

### Design of the glycan engineered SARS-CoV-2 RBD antigens – M7 and M8

**Figure 1:**
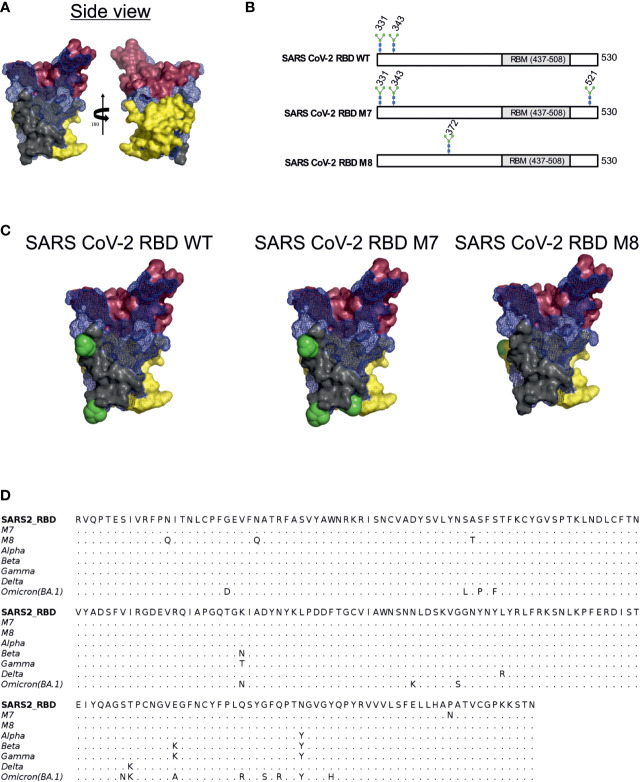
Rational immunogen design of glycan engineered SARS CoV-2 RBD mutants. **(A)** Mesh surface representation of SARS CoV-2 RBD protein. The epitope recognised by B38 is represented in red-brown solid surface, CR3022 in yellow solid surface, and S309 in grey solid surface. **(B)** The glycan sites in wild-type SARS-CoV-2 RBD, M7 and M8 designs are highlighted by the stick representation of sugar molecule. **(C)** Surface representation of wild-type SARS-CoV-2 and glycan engineered M7 and M8 with glycans shown as green spheres. **(D)** Multiple sequence alignment of RBD WT with M7, M8, and VOCs.

Glycan engineering of antigenic epitope regions has been shown to focus and facilitate the induction of immune responses to certain epitopes and enhance the elicitation of neutralising antibodies by either shielding of non-neutralising epitopes and/or exposing and focusing antibodies to conserved neutralising epitope-rich regions (22). To design modified SARS-CoV-2 RBD antigens, we added or removed N-linked glycosylation sites to the SARS-CoV-2 RBD (WA-1/2020) to mask or expose epitopes described by defined mAbs. Three epitope regions of the class 1 monoclonal antibody (mAb) B38 (57) (**Figure 1A**, shown in red brown), class 3 mAb CR3022 (41) (**Figure 1A**, shown in yellow) and class 4 S309 (42) (**Figure 1A**, shown in grey) were selected for glycan masking. The epitope regions of the mAb CR3022 and mAb S309 are outside of the SARS-CoV-2 receptor binding motif (RBM) which is known to be recognised by many antibodies in convalescent sera from SARS-CoV-2 infected individuals (58), while the epitope region of B38 overlaps with the RBM. The S309 mAb has been shown to bind and neutralise SARS-CoV-1 and SARS-CoV-2, whereas the CR3022 mAb will bind to the RBD of both spikes, but only neutralises SARS-CoV-1 (41). The S309 binding epitope has two naturally occurring N-linked glycosylation sites at position 331 and 343 (**Figures 1B, C**), while the CR3022 epitope site is devoid of any glycan. Interestingly, the CR3022 epitope has one glycosylation site in SARS-CoV-1. To understand the effect of glycosylation modifications on the overall immune response to SARS-CoV-2 RBD, two SARS-CoV-2 glycan mutants, namely SARS-CoV-2 RBD M7 (henceforth referred as M7) and SARS-CoV-2 RBD M8 (henceforth referred as M8) (**Figures 1B, C**) were engineered. In M7, an additional glycan was added at position 521(P521N) located in the epitope region of CR3022 (**Figures 1B, C**). The SARS-CoV-2 RBD M8 was engineered by removing the two natural glycans at position 331(N331Q) and 343 (N342Q) located in the S309 epitope and addition of a glycan at position 370 by introducing mutation A372T that is known to be present in the CR3022 epitope of SARS-CoV-1 (**Figures 1B, C**). Multiple sequence alignment of SARS-CoV-2 RBD of WA-1/2020, Alpha, Beta, Gamma, Delta, and Omicron (BA.1) with M7 and M8 shows that the mutations introduced in these two designs have not yet been observed in any of the VOCs (**Figure 1D**).

### M7 DNA based vaccine candidate favourably tips the ratio of neutralising antibodies to binding antibodies against SARS-CoV-2

**Figure 2:**
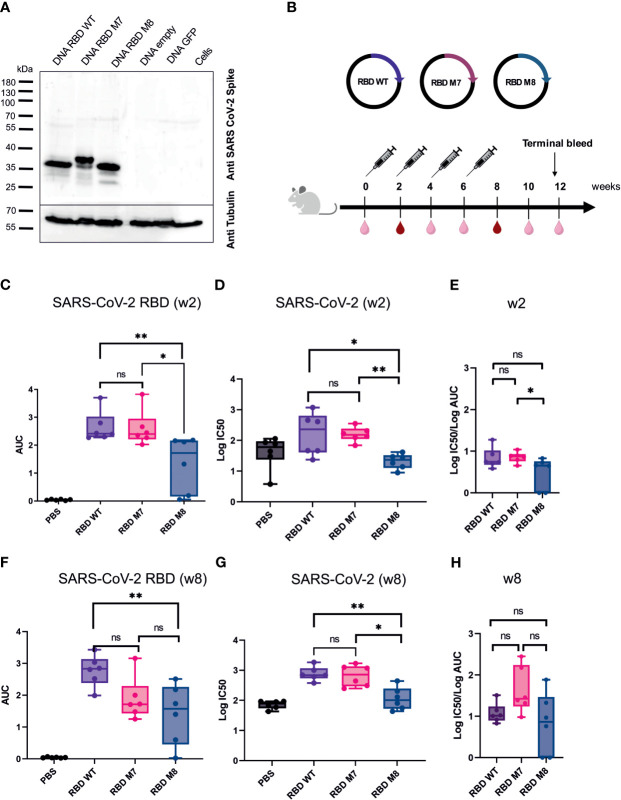
SARS CoV-2 RBD DNA-based vaccine candidates induce humoral immune response in Balb/c mice. **(A)** Expression analysis of DNA based vaccine candidates encoding glycan engineered SARS CoV-2 RBD mutants by Western blot of transfected HEK293T cells harvested after 48 h. Size in kilodaltons **(kDa)** and size of the molecular weight marker are indicated. The “DNA empty” lane refers to cells transfected with the same vector but missing the gene insert, the “Cells” lane represents untransfected cells.**(B)** Immunization schedule of BALB/c mice (n=6) vaccinated four times with DNA-based vaccines encoding SARS CoV-2 RBD WT, M7 and M8, respectively. Bleeds were taken at two-week intervals (light right symbol) and bleeds at week 2 and 8 (dark red symbol) were anlaysed for humoral repsonse. Binding antibody titers **(C)** represented as area under the curve (AUC) and neutralizing antibodies **(D)** shown as logIC50 values against SARS CoV-2 two weeks after the first DNA immuniztation. **(E)** Ratio of log IC50 and log (AUC) was calculated to capture the proportion of neutralising antibodies to binding antibodies 2 weeks after the first DNA immunization. Binding **(F)** and neutralizing antibodies **(G)** induced after 4 immunisations with the respective SARS CoV-2 RBD DNA vaccine candidate. **(H)** Calculated ratio of log IC50 and log(AUC) at week 8 after 4 DNA immunizations. The Mann-Whitney statistical test was applied (*p < 0.05; **p < 0.005 as asteriks or ns for non-significant).

For *in vitro* characterisation of the DNA-based glycan engineered M7 and M8, total cell lysates from HEK293T cells were prepared 48 h after transfection, followed by Western blot analysis. Staining of the membrane with a polyclonal SARS-CoV-2 rabbit antibody showed that all the DNA constructs were successfully expressed at the expected band of approximately of 35 kDa. M7 appears in the immunoblot blot slightly higher due to the addition of a glycan, whereas M8 runs slightly lower due to the removal of glycosylation sites compared to the SARS-CoV-2 RBD wt protein (**Figure 2A**). To evaluate the immunogenicity of the DNA vaccine candidates M7 and M8 in comparison to the original SARS-CoV-2 vaccine strain based on the original WA-1/2020 sequence, BALB/c mice (n=6) were vaccinated with 50 µg of the DNA vaccine construct expressing M7, M8 or wild type SARS-CoV-2 RBD subcutaneously four times at two-week intervals (**Figure 2B**). An overview of the SARS-CoV-2 RBD DNA vaccine constructs including the mutations for each construct is provided in **Table 1**. Blood samples were collected every two weeks and analysed for both binding antibodies (bAb) and neutralising (nAb) using SARS-CoV-2 RBD based direct ELISA and pseudovirus neutralisation assay against SARS-CoV-2, respectively.

**Table 1:** Glycan engineered SARS-CoV-2 RBD DNA vaccine constructs evaluated in this study.

DNA vaccine construct	Mutations
pURVac SARS-CoV-2 RBD wt	–
pURVAc SARS-CoV-2 RBD M7	P521N
pURVac SARS-CoV-2 RBD M8	N331Q N343Q A372T

After the first DNA immunisation (2 weeks post immunisation), no significant difference was observed in the levels of bAb (**Figure 2C**) and nAb titres (**Figure 2D**) induced by the M7 and RBD wt vaccine construct, whereas M8 elicited weaker bAb and nAb responses in comparison to both M7 and wt SARS-CoV-2 RBD (**Figures 2C, D**). Interestingly, after the fourth and last DNA immunisation, mice immunised with M7 generated significantly lower bAb titre (p=0.04) in comparison to sera after first immunisation, while M8 generated comparable bAb titre after first and last immunisation (**Supplementary Figure 1A**). However, nAb titres were significantly increased for both RBD M7 (p=0.009) and RBD M8 (p=0.002) after four immunisations in comparison to first immunisation, indicating the impact of four DNA immunizations and affinity maturation (**Supplementary Figure 1B**). Both bAb and nAb titres for M7 were comparable to RBD wt after four immunisations (**Figures 2F, G**), while M8 generated substantially lower bAb (**Figure 2F**) and nAb (**Figure 2G**) in comparison to wt SARS-CoV-2 RBD but comparable bAb to M7. We calculated the ratio of IC_50_ and AUC values, to enumerate the proportion of nAb for a given bAb titre. The observation of different ratios of bAb and nAb between M7, M8, and wt SARS-CoV-2 RBD at week 2 (**Figure 2E**) and week 8 (**Figure 2H**), suggest that masking of the CR3022 epitope *via* the addition of a glycan at position 521 induces a trend towards a greater proportion of neutralising antibodies for a given bAb titre while the de-masking of S309 epitope by removing the glycan position at 331 and 343 and simultaneous introduction of glycan at position 372, reduces both the bAb as well as nAb. Taken together, the SARS-CoV-2 RBD wt construct induced homologous bAbs, whereas the SARS-CoV-2 RBD M7 was capable to elicit heterologous bAbs and therefore to focus and direct immune response to the neutralising epitopes through shielding of the CR3022 epitope. As the M8 construct elicited weaker bAbs (**Figure 2F**) and nAbs (**Figure 2G**) the construct was excluded from further studies.

### Design, generation and biochemical characterisation of recombinant MVAs expressing M7 and wt SARS-CoV-2 RBD

**Figure 3:**
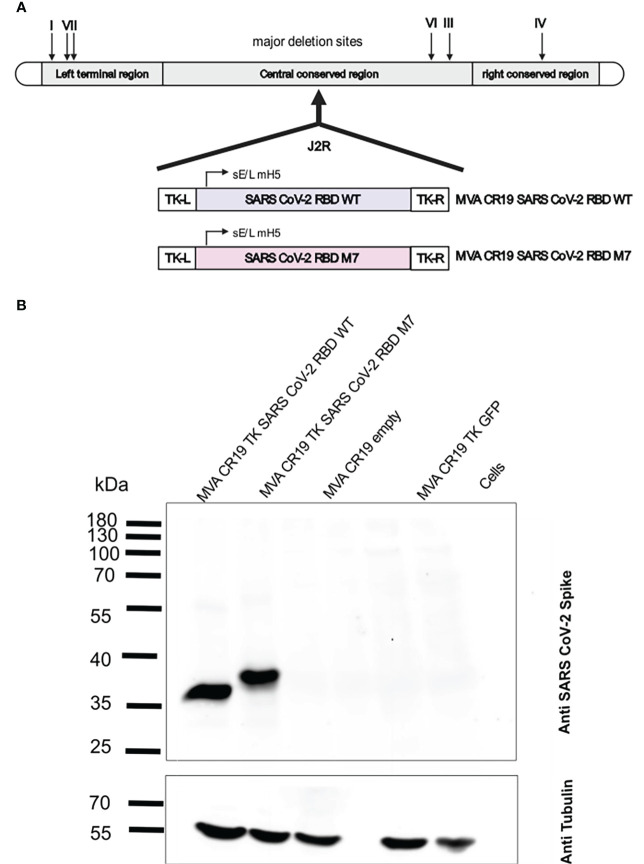
Construction and biochemical characterisation of recombinant MVAs encoding for SARS CoV-2 RBD WT and SARS CoV-2 RBD M7 antigens. **(A)** Schematic representation of the MVA genome and design of the recombinant SARS CoV-2 RBD WT and SARS CoV-2 RBD M7 MVAs. The J2R region or TK locus was used to insert the antigens for SARS CoV-2 RBD WT and SARS CoV-2 RBD M7 *via* homologous recombination. **(B)** Western blot analysis of recombinant MVAs encoding SARS CoV-2 RBD WT and SARS CoV-2 M7 RBD using HEK293T cell lysates infected with a MOI of 2.0 and harvested after 24 h. Size in kilodaltons **(kDa)** and corresponding bands of the protein standard are indicated. The “MVA CR19 empty” lane represents cells infected with the same MVA construct, but lacking the antigen gene in the backbone, the “Cells” lane represents uninfected cells.

Since MVA as a recombinant viral vector is known to effectively boost DNA-primed specific immune responses against multiple infectious diseases (59, 60), recombinant MVAs were generated encoding the SARS-CoV-2 wt RBD and M7. (**Figure 3A**). The antigens were integrated into the TK locus of the CR19 MVA genome (**Figure 3A**). MVA CR19 is a novel, genetically stable MVA strain that replicates to very high titres (in the range of 10^9^ IU/mL) in the AGE1.CR.pIX production cell line (54). Compared to wild-type MVA, CR19 releases a larger number of infectious particles into the culture supernatant resulting in higher yields (53). MVA-CR19 is furthermore genetically characterized by a recombination where the left terminal region is replaced by the right terminal region. The result is an expansion of terminal homology from 15 kb to 27 kb, loss of three genes previously encoded in the left and duplication of the gene dosis for 9 genes encoded in the right ITR. Expression of the antigens was tested by Western blot analysis in HEK293T cells infected with MVA CR19 TK SARS-CoV-2 wt RBD and MVA CR19 TK M7 at a MOI of 2. Cells were lysed for expression analysis 24 h post infection. The immunoblot stained with a polyclonal SARS-CoV-2 S specific rabbit antibody revealed good antigen expression of both recombinant MVAs with a band around 35 kDa for MVA CR19 TK SARS-CoV-2 RBD wt and a slightly larger band for the glycan engineered MVA CR19 TK M7 (**Figure 3B**).

### M7 DNA prime followed by a MVA boost induces higher and longer lasting cross-reactive titres binding and neutralizing antibodies against VOCs

**Figure 4:**
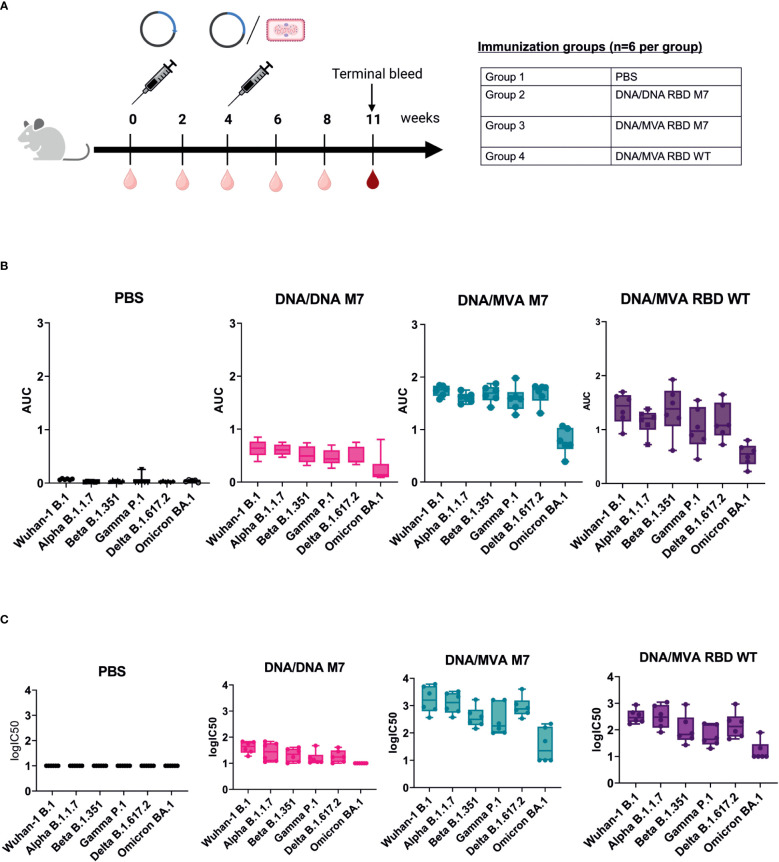
DNA/MVA superior to DNA/DNA regimen in induction of binding and neutralising antibodies against VOCs. **(A)** Immunization schedule of BALB/c mice vaccinated with either DNA/DNA or DNA/MVA regimen. For the DNA immunization, mice (n=6) received 50 µg DNA vaccine subcutaneuosly, whereas for the MVA boost mice were immunized either with SARS CoV-2 RBD WT or SARS CoV-2 RBD M7 MVA at a dose of 2x10^7^ pfu. Bleeds were taken at two-week intervals (light red symbol) and the terminal bleed at week 11 (dark red symbol) was analysed. **(B)** Binding antibody titers against VOCs represented as AUC values measured in mice sera from week 11. **(C)** Neutralization titers shown as logIC50 against VOCs in mice sera collected at week 11.

To evaluate whether a heterologous DNA prime/MVA boost regimen can induce higher and long-lasting broadly neutralising antibodies against VOCs, BALB/c mice (n=6) were immunised subcutaneously with 50 µg of DNA vaccines encoding SARS-CoV-2 RBD wt or SARS-CoV-2 RBD M7 on day 0. At week 4, the mice were either immunised subcutaneously with 50 µg of DNA vaccines encoding SARS-CoV-2 RBD wt or SARS-CoV-2 RBD M7 or were vaccinated intramuscularly with a heterologous MVA boost using MVA SARS-CoV-2 RBD wt or MVA SARS-CoV-2 RBD M7 at with a dose 2x 10^7^ pfu per animal, respectively. The bleeds were collected 2 weeks after each immunisation. To evaluate longevity and durability of binding and neutralising antibodies after homologous and heterologous prime/boost immunisation, the terminal bleed at week 11 was analysed (**Figure 4A**). Therefore, the sera were tested by direct RBD ELISA regarding the induction of bAbs against WA-1/2020 B.1, Alpha B.1.1.7, Beta B.1.351, Gamma P.1, Delta B.1.617.2, and Omicron BA.1 RBD. Interestingly, the titres of anti-SARS-CoV-2 RBD binding antibodies at week 11 were significantly higher across all the tested VOCs in mice that received a boost with MVA (**Figure 4B**, **Supplementary Figure 2**). Comparing MVA SARS-CoV-2 RBD wt and RBD M7 across all VOCs, significantly higher binding antibody titres are observed for RBD M7 for WA-1/2020 (p=0.02), Alpha B.1.1.7 (p=0.002), Gamma P.1 (p=0.02) and Delta B.1.617.2 (p=0.02) (**Figure 4B**, **Supplementary Figure 2**), while comparable binding antibody titres are observed for Beta B.1.351 and Omicron BA.1.

To measure the impact of a heterologous DNA prime/MVA boost immunisation on the induction of higher, and long lasting broadly neutralising antibody, mice sera from week 11 were evaluated against WA-1/2020 B, Alpha B.1.1.7, Beta B.1.351, Gamma P.1, Delta B.1.617.2 and Omicron BA.1 using lentiviral pseudotype microneutralisation assays. The neutralising antibody response showed same trend as the binding antibody levels measured by direct RBD ELISA, with a significant increase for mice that received a heterologous MVA boost versus mice that were vaccinated two times with DNA vaccine (**Figure 4C**, **Supplementary Figure 3**). Higher nAb titres were observed for WA-1/2020, Gamma, and Delta for MVA M7 in comparison to MVA wt but comparable nAb were observed for Alpha, Beta, and Omicron (**Supplementary Figure 3**). A log fold decrease in the both the bAb and nAb for omicron is expected due to the high number of mutations in RBD region of omicron including the S309 and CR3022 epiotopes.

In conclusion, SARS-CoV-2 RBD M7 DNA prime followed by a MVA boost was clearly superior to two times DNA immunisation and induced higher and more cross-reactive titres binding and neutralising antibodies against all the tested VOCs and were still relatively high 7 weeks after the MVA boost.

### DNA-MVA prime-boost regime reduces viral load after challenge with SARS-CoV-2 wildtype strain

**Figure 5:**
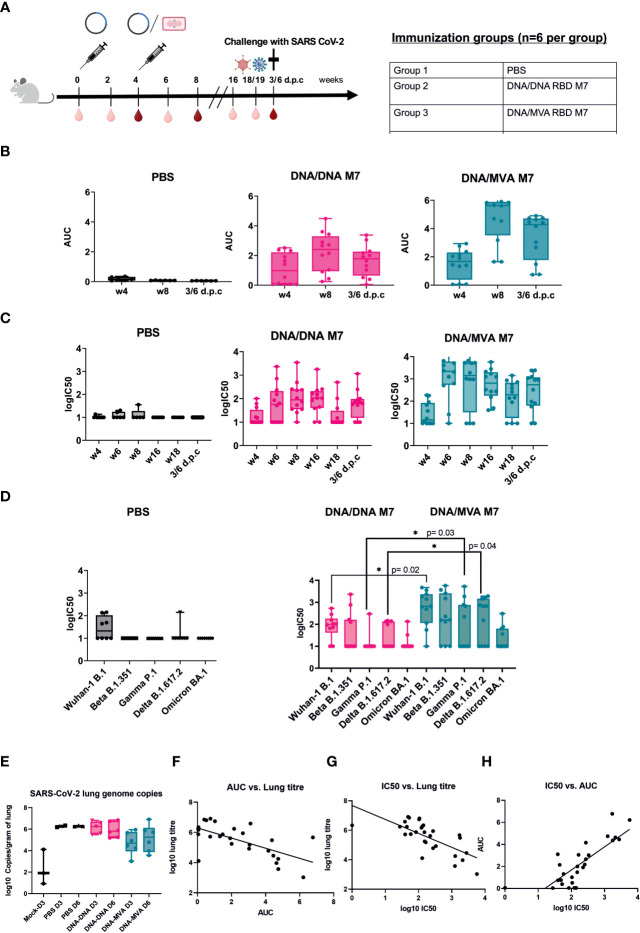
Challenge in human ACE2 transduced mice with SARS-CoV-2 wildtype virus. **(A)** Immunization schedule of BALB/c mice vaccinated (n=12) using different DNA prime/MVA boost regimen followed by a challenge with SARS-CoV-2 live virus. For challenge, BALB/c mice were transduced with 1x10^7^ pfu of the ad5-huACE2 vector five days before infection with 1x10^4^ pfu Australia/VIC01/2020 SARS-CoV-2 virus. Bleeds were taken in two-week intervals (light red symbol) until week 8 and then again at week 16, 18/19 and 6 days post challenge (6 d.p.c). Bleeds at week 4, 8 and 6 d.p.c were analysed (dark red symbol). Binding antibody titers (AUC) **(B)** and neutralising antibodies (logIC50) **(C)** against SARS-CoV-2 were analyzed 4 weeks after prime and boost respectively and 6 days post challenge (d.p.c). **(D)** Neutralisation titers (logIC50) against all circulating VOCs to date analyzed in mouse sera obtained after 6 d.p.c. **(E)** SARS-CoV-2 genome copies from the lungs of infected mice at day 3 (D3) and day 6 (D6) post infection shown as log10 copies/gram of lung. **(F–H)** Correlation of binding (AUC) and neutralising (logIC50) antibody titer versus lung titre.

To investigate whether a homologous SARS-CoV-2 RBD M7 DNA prime/DNA boost or heterologous SARS-CoV-2 RBD M7 DNA prime/MVA boost regimen can provide protection from SARS-CoV-2 wild type live virus, a challenge study using human ACE2 transduced BALB/c mice was carried out. For immunisation one group of BALB/c mice (n=12) received two doses of 50 µg of the SARS-CoV-2 M7 DNA vaccine subcutaneously, whereas another group of BALB/c mice (n=12) was vaccinated using a heterologous SARS-CoV-2 RBD M7 DNA prime/MVA boost vaccination regimen with 2x10^7^ pfu (plaque forming unit) intramuscularly at day 0 and week 4. The study was set up longitudinally and sera were collected 2 weeks after each immunization, followed by week 16 and 18 and the terminal bleed 6 days post challenge (d.p.c) (**Figure 5A**).

To confirm the durability and waning immunity over time, sera from the longitudinal challenge study were analysed for their binding and neutralising capacity across all variants. After the prime immunisation with DNA, bAb responses were detected in 7/12 mice in the DNA/DNA group whereas 9/12 mice in the DNA/MVA group showed binding antibodies against SARS-CoV-2 (**Figure 5B**). The neutralising antibody response against SARS-CoV-2 was low after priming with DNA (**Figure 5C**). After the boost with either DNA or MVA, both the binding and neutralising antibodies increased with a steady level of nAb till week 16, following which a drop in nAb is observed (**Figures 5B, C**). As previously, both bAb (**Figure 5A**) (p=0.014) and nAb responses (**Figure 5B**) (p=0.003) were significantly higher in the MVA boosted group compared to the DNA boosted group at week 6.

Mice were rendered susceptible to SARS-CoV-2 by intranasal administration of Ad5-huACE-2 construct and challenged five days later with 1x10^4^ pfu SARS-CoV-2 Australia/VIC01/2020. Due to the lack of published disease readouts in wild type mice at the time of challenge, even after Ad5-huACE-2 transduction, the decision was made to cull mice at days 3 and 6 post infection to measure virus replication in the lungs. An increase in nAb titres was observed in the terminal bleed sera, in line with a typical reaction to encountering the virus (**Figure 5C**). Terminal sera from mice were also tested against the SARS-CoV-2 VOCs - Beta, Gamma, Delta, and Omicron, with a subset of mice showing decreases in or abrogation of nAb, as expected based on the published literature, particularly to Gamma and Omicron (**Figure 5D**). In mice that received MVA boost, a significant increase in nAbs could be observed after challenge for WA-1/2020 \(p=0.02), Gamma P.1 (p=0.03) and Delta B.617.2 (p=0.004) compared to animals that were vaccinated twice with DNA (**Figure 5D**). As binding, and neutralising antibodies across all VOCs could be detected 14 weeks after the last immunisation and even just 1 week before challenge at week 18 (**Figure 5C**) suggested that the MVA boost induces a strong, broad, and longer lasting neutralising antibody response.

Mice that received a heterologous MVA boost showed some reduction of viral load in the lungs after challenge. In contrast, the mice that received DNA boost did not show any reduction of SARS-CoV-2 lung genome copies when compared to naïve controls (**Figure 5E**). An inverse correlation was observed between copies of SARS-CoV-2 in the lungs of infected mice and their respective bAb (**Figure 5F**) or nAb (**Figure 5G**) antibody titre (Pearson’s r2= -0.49 and -0.62 respectively, p=<0.0001), confirming a correlation between RBD-directed neutralising antibodies and the reduction of SARS-CoV-2 replication in the lungs. Challenged mice also showed a weak positive correlation between detected nAb and bAb responses (r2 = 0.44, p=<0.0001) (**Figure 5H**). These results confirm that neutralising antibodies generated in mice immunised with the glycan engineered M7 vaccine reduce viral load in the lungs in BALB/c mice *in vivo*.

## Discussion

The ongoing COVID-19 pandemic is characterized by emergence of new SARS-CoV-2 VOC that are highly transmissible and able to escape pre-existing antibodies. At the same time, the risk of breakthrough infections is estimated to increase with waning immune efficacy approximately 6 months after vaccination or natural infection. Periodic boosters are therefore recommended to disrupt evolution and spread of new waves of variants. Given these issues, improved next generation vaccine candidates are required that provide longer lasting immunity and better coverage to known as well as emerging variants. Here, we present pre-clinical proof-of concept data demonstrating that a novel glycan engineered RBD based vaccine antigen generated a higher magnitude of neutralising and binding antibodies to a broad panel of SARS-CoV-2 spikes compared to wt SARS-CoV-2 RBD as antigen. We generated two glycan site modified SARS-2 RBD *viz.* M7 and M8. In M7, a glycan site is introduced in the epitope region of a non-neutralising antibody (CR3022). In M8, glycan sites are removed from the epitope region of a neutralising antibody (S309) and a glycan site reported in SARS-CoV-1, different from M7, is introduced in the epitope region of the non-neutralising antibody CR3022. Results obtained here indicate that M7 generated a higher proportion of neutralising antibodies in comparison to wt and M8. After four successive immunisations, M7 and M8 generated a similar binding titre of binding antibodies but substantially different levels of neutralising antibodies. We hypothesize that the superior magnitude and quality of the response to M7 is due to the introduction of a steric hindrance for binding of CR3022 and related epitope-sharing non-neutralising antibodies. Decreased binding efficacy may prevent affinity maturation and clonal selection of antibodies that bind the antigen but do not contribute to neutralisation. An opposing mechanism may determine immune responses to M8. The glycosylation sites that were removed in M8 are part of a neutralizing epitope region. Interaction with S309 and related epitope-sharing antibodies may thus be disrupted, reducing the efficacy of affinity maturation and clonal expansion for neutralising antibodies. This observation suggests that de-glycosylation of the neutralising epitope leads to an inferior vaccine construct in case of SARS-CoV-2. To further interrogate the superiority of M7 in comparison to the wt SARS-CoV-2 sequence based vaccines, we tested and compared the immunogenicity of M7 in a DNA-DNA versus a DNA-MVA prime-boost regime. MVA is well established as an excellent boosting vaccine vector following a DNA priming immunisation (59, 60). DNA-MVA prime-boost regime induces significantly higher and durable binding as well as neutralising antibodies titres in comparison to same modality prime-boost regimes. Immunisation with M7 in DNA-MVA prime-boost regime demonstrated better neutralisation across all the VOCs tested. The observation of weaker Omicron neutralisation is in line with previously published data. Based on all these observations, we propose that the better neutralisation ability against VOCs by M7 is due to the higher proportion of the neutralising antibodies recruited in comparison to wt SARS-CoV-2 immunisation. A reduced viral load in human ACE2 transduced mice was observed following M7-DNA prime/M7-MVA boost as compared to M7-DNA/M7-DNA regimen.

These data provide pre-clinical evidence of the superiority of the M7 modified vaccine antigen over the wt SARS-CoV-2 across two vaccination platforms: DNA-DNA and DNA-prime, MVA-boost protocols. Demonstration of enhanced neutralising and binding antibody titres, and a broader immune response to neutralizing epitopes by introduction of a specific glycosylation motif provides important proof of concept for this type of modification of vaccine antigens.

## Data availability statement

The raw data supporting the conclusions of this article will be made available by the authors, without undue reservation.

## Ethics statement

The animal study was reviewed and approved by AWERB, University Biomedical Services, University of Cambridge.

## Author contributions

Conceptualization, JH, RW, RK. Data curation: GC, MB, SV. Investigation: GC, MB, SV, BA, JH, RK, RW. Methodology: GC, MB, SV, HW, AS, MSS, CG, JDR, ATS, AN, CH, DW, MF, MP, PT, DP. Critical reagents: SE, PN, DC, NT, IJ, VS. Project administration: JH, RW. Resources: JH, RW. Supervision: JH, RW, RK. Visualization: GC, MB, SV. Writing—original draft: GC, MB, SV. Writing— review and editing: GC, MB, SV, JH, RW, IJ. All authors contributed to the article and approved the submitted version.
