# Supplementary material for: Glycan masking of a non-neutralising epitope enhances neutralising antibodies targeting the RBD of SARS-CoV-2 and its variants
Source: Front Immunol. 2023 Feb 23;14:1118523. doi: 10.3389/fimmu.2023.1118523 (PMC9995963; doi:10.3389/fimmu.2023.1118523)
Supplement: Supplementary file 1 [file DataSheet_1.docx]

Supplementary Material

Glycan masking of a non-neutralising epitope enhances neutralising antibodies targeting the RBD of SARS-CoV-2 and its variants.

## Supplementary Figures


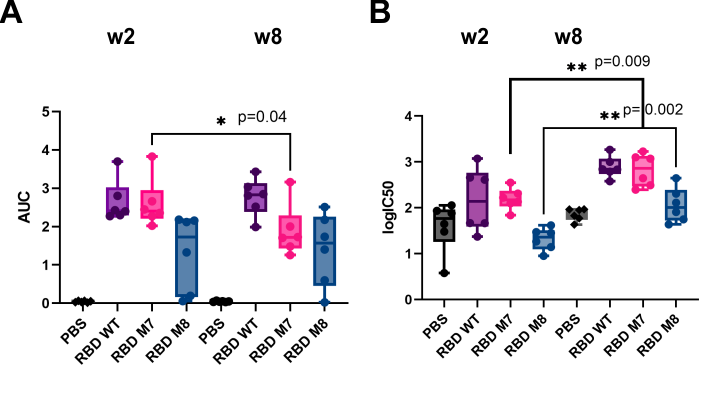


**Supplementary figure 1. Impact of DNA immunizations on the induction of binding and neutralising antibodies of RBD M7 and M8.** (A) Binding antibody titers (AUC) of mice (n=6) that received one (w2) or four DNA immunizations (w8) of DNA RBD WT, M7 or M8. Sera were collected at week 2 and 8 and analysed by ELISA against SARS CoV-2 RBD. (B) Neutralizing antibody titers against SARS-CoV-2 shown as IC_50_ values determined from sera taken at week 2 after one DNA immunization or week 8 after 4 DNA immunizations. The Mann-Whitney statistical test was applied (*p < 0.05; **p < 0.005 as asteriks or ns for non-significant).


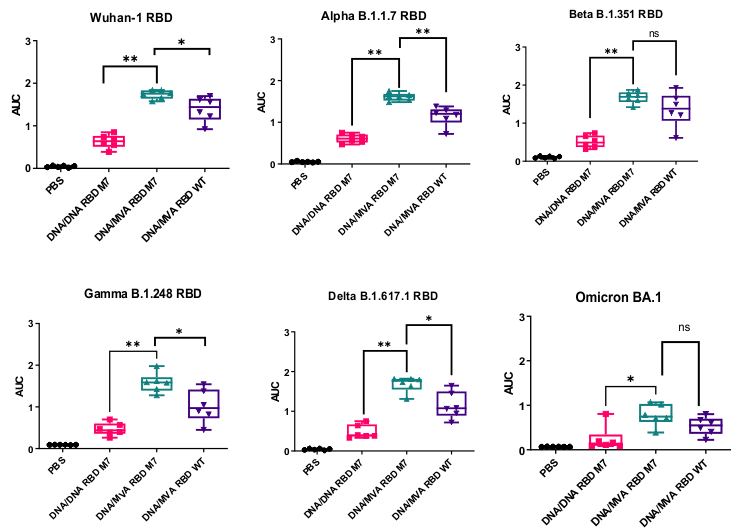


**Supplementary figure 2. Heterologous DNA prime/MVA boost immunisation induces significantly elevated binding antibodies across all VOCs.** Binding antibody titers of mice (n=6) that received either DNA RBD M7 or MVA RBD M7 or MVA RBD WT as boost following homologous vaccine design as DNA prime were determined by ELISA against different VOCs at week 11. The Mann-Whitney statistical test was applied (*p < 0.05; **p < 0.005 as asteriks or ns for non-significant).


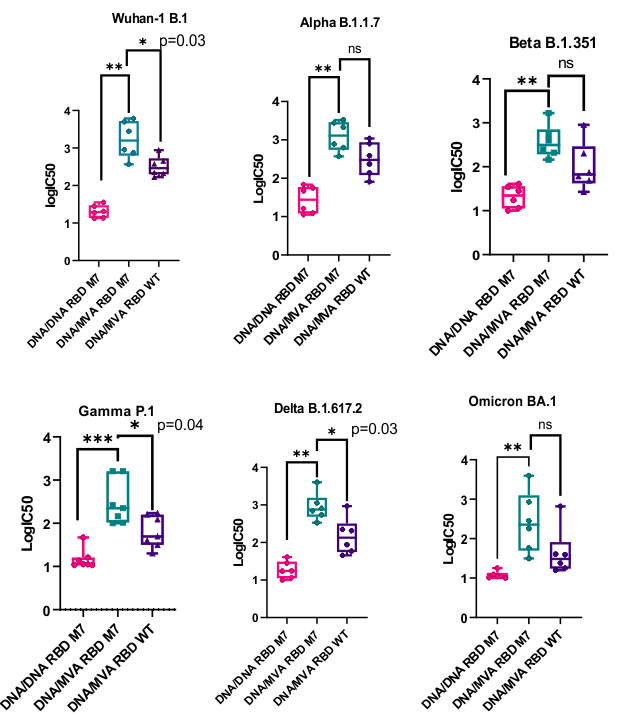


**Supplementary figure 3. Heterologous DNA prime/MVA boost immunisation significantly enhance neutralizing antibodies across all VOCs.**Neutralization titers (logIC50) against all VOCs were analyzed in mice (n=6) that received either a homologous or heterologous MVA RBD M7/RBD WT boost immunisation at week 11. The Mann-Whitney statistical test was applied (*p < 0.05; **p < 0.005 as asteriks or ns for non-significant).
